# Impact of intracardiac echocardiography on radiofrequency catheter ablation for atrial fibrillation: a clinical study on procedural parameters and post-procedural recurrence

**DOI:** 10.3389/fcvm.2026.1746680

**Published:** 2026-05-18

**Authors:** Feiyue Liu, Maojing Wang, Yuefeng Jv, Zhihui Wang, Shanglang Cai

**Affiliations:** 1 First Clinical Medical College, Qingdao University, Qingdao, China; 2 Department of Cardiology, The Affiliated Hospital of Qingdao University, Qingdao, China; 3 Qingdao Medical College, Qingdao University, Qingdao, China; 4 Department of Internal Medicine, The Affiliated Hospital of Qingdao University, Qingdao, China

**Keywords:** atrial fibrillation, first-pass isolation, intracardiac echocardiography, procedural parameters, radiofrequency catheter ablation, recurrence

## Abstract

**Objective:**

Radiofrequency catheter ablation (RFCA) has become a first-line treatment for atrial fibrillation (AF). Abnormal force-over-time (FOT), a procedural parameter, leads to gaps and influences ablation continuity and transmurality. Intracardiac echocardiography (ICE) provides valuable guidance during RFCA. This retrospective study investigates the impact of ICE on FOT and recurrence in RFCA.

**Methods:**

Propensity score matching (PSM) was performed to mitigate the impact of intergroup imbalance. The Kendall correlation, point-biserial correlation coefficient test and multivariate Gamma regression were employed to analyze factors influencing procedure and ablation time. Univariate and multivariate Cox regression analysis were performed to analyze factors influencing recurrence.

**Results:**

A total of 167 patients were enrolled. After PSM, 102 patients were included with 51 patients in each of the ICE and non-ICE groups. After adjusting for confounding factors, baseline eGFR (HR = 0.97, 95% CI 0.94–0.99, *P* = 0.017) and high FOT count in RPS segment (HR = 1.48, 95% CI 1.05–2.09, *P* = 0.025) were independent factors influencing recurrence. The eGFR (RR = 1.005, 95% CI 1.000–1.009, *P* = 0.038) and low FOT count in LAI segment (RR = 1.019, 95% CI 1.002–1.036, *P* = 0.033), high FOT count in RPI segment (RR = 0.948, 95% CI 0.902–0.996, *P* = 0.036) were factors influencing ablation time, while high FOT count in LF segment (RR = 1.072, 95% CI 1.008–1.139, *P* = 0.029), cardiomyopathy (RR = 2.181, 95% CI 1.339–3.554, *P* = 0.002), and LAAO (RR = 1.485, 95% CI 1.249–1.766, *P* < 0.001) were factors influencing procedure time.

**Conclusions:**

ICE influences the distribution of abnormal FOT across PV segments. ICE-guided procedures demonstrated improvement in recurrence. However, the benefits may not be directly provided by ICE but rather mediated through the occurrence of abnormal FOT.

## 1 Introduction

Atrial fibrillation (AF) is one of the most common arrhythmias affecting approximately 37.6 million people globally, with a prevalence of 0.51%, and is closely associated with stroke, heart failure (HF) renal dysfunction and other complications (1–3). Catheter ablation (CA), including radiofrequency catheter ablation (RFCA), can restore sinus rhythm and is recommended as a first-line therapy (4). The clinical characteristics of AF exhibit complexity and heterogeneity. Extensive prior research has focused on various aspects of AF management, including anticoagulation strategies, medication adjustments, and genetic susceptibility (2, 5–8). Numerous clinical risk scores have been developed to predict AF prognosis (6). In recent years, the application of machine learning algorithms has introduced novel approaches for achieving more precise prediction (9). The success and safety of RFCA depend on precise anatomical localization and complication monitoring. AF recurrence remains a significant concern, with freedom from recurrence at one year after the first procedure ranging only from 60% to 80%, lower in patients with persistent AF, significant structural heart disease or multiple comorbidities (10). To improve acute success and reduce recurrence safely and efficiently, previous research has explored parameters like contact force (CF), ablation power and ablation duration (11). It is recognized that target ablation values should vary by anatomical location, and the distributions of gaps, re-ablation sites and recurrence sites differ across segments (12, 13). A contiguous, transmural ablation without electrical gaps is crucial for achieving immediate termination of AF and ensuring long-term freedom from recurrence. The force-over-time (FOT) parameter represents the proportion of time during which the catheter maintains contact force throughout the ablation of lesions. Excessively high or low FOT values can adversely affect the width and depth of the ablation lesion. Abnormal FOT leads to gaps and reduced first-pass isolation (FPI), influencing contiguity and transmurality of the ablation lines. In previous studies, FOT was pre-set before the procedure and was often overlooked in subsequent analysis. In recent years, studies showed that the use of echocardiography (ICE) in RFCA has benefits for both patients and operators. ICE guidance can reduce procedure time, ablation time, transseptal puncture (TS) time and major peri-procedural complications such as cardiac tamponade. Compared to traditional fluoroscopy-guided RFCA, ICE reduces learning curve for RFCA, fluoroscopic time and radiation exposure (14–22). Compared with conventional fluoroscopy, ICE provides real-time, intuitive imaging that enhances procedural safety and efficacy and potentially offering additional assistance in guiding transseptal puncture site adjustment and catheter contact. This study investigates the impact of ICE guidance on RFCA outcomes, including first-pass isolation (FPI), FOT, procedure duration, ablation time, and postoperative recurrence. The findings aim to inform the development of a clinical predictive model for real-time efficacy and recurrence risk in ICE-guided RFCA for AF.

## 2 Methods

### 2.1 Study population

This study retrospectively enrolled patients with AF who were hospitalized and underwent RFCA at the Shinan Campus of the Affiliated Hospital of Qingdao University between June 2022 and June 2023. Inclusion Criteria: (1) Pre-procedural diagnosis of AF by routine ECG or Holter ECG, including patients with paroxysmal AF (PAF) and persistent AF (PeAF). (2) Presence of procedure indications and performance of RFCA during hospitalization. (3) Age between 18 and 85 years. (4) Signed informed consent for the procedure. Exclusion Criteria: (1) Previous history of CA. (2) Uncontrolled thyroid dysfunction during hospitalization, with T3 or T4 levels outside the normal range. (3) Concomitant severe valvular heart disease, including severe valvular regurgitation or stenosis, not treated surgically. (4) Concomitant renal disease during hospitalization requiring dialysis treatment. (5) Concomitant severe liver function impairment or coagulation dysfunction. (6) Concomitant bradyarrhythmia, such as AF with long R-R interval or sick sinus syndrome (SSS), requiring artificial cardiac pacemaker implantation. (7) Concomitant malignant tumors or other diseases likely to affect prognosis, resulting in life expectancy under one year. (8) Procedure cancellation due to patient's refusal or other reasons pre-procedurally. (9) Inability to match patient information in the CARTO system with data in the Hospital Information System (HIS) due to incomplete annotation of patients' details. Collected data comprised demographics (age, sex, height, weight), medical history, comorbidities, CHA₂DS₂-VASc score, routine laboratory tests, standard and 24 h Holter ECG, chest CT, peripheral vascular ultrasound, and echocardiography. All patients received continuous oral anticoagulant therapy for at least three weeks prior to the procedure and for up to four weeks post-procedure. Baseline characteristics were extracted from the HIS to ensure comprehensive documentation of patient profiles.

### 2.2 Radiofrequency ablation

After ICE was introduced at this center, the decision to use ICE during the procedure was made based on the operator's judgment and the patients' pre-procedural preference. The workflows of RFCA with and without ICE guidance are described as follows. For procedures without ICE assistance, patients were placed in the supine positions. Under local anesthesia with lidocaine, the right femoral vein was punctured and a 6F sheath was inserted. A second puncture was performed to place an 8.5F Swartz sheath, which was advanced into the superior vena cava. The atrial septum puncture needle was then guided along the sheath to the fossa ovalis (FO), and a transseptal puncture was performed. The Swartz sheath was advanced into the left atrium (LA), through which a star-shaped mapping electrode was introduced. Pulmonary angiography was performed, and a left atrial model was established under the Carto system. Electrical isolation of the bilateral pulmonary vein antra (PVA) was carried out. After a 10 min observation period, the star-shaped mapping electrode was sequentially placed in the right superior PV (RSPV), right inferior PV (RIPV), left superior PV (LSPV), and left inferior PV (LIPV) to confirm bidirectional electrical isolation. For patients undergoing ICE-guided procedures, a 10F sheath was inserted, and the ICE catheter was advanced into the right atrium (RA) via the sheath. The ICE was rotated clockwise to sequentially image the tricuspid valve, right ventricle (RV), aortic valve, coronary sinus (CS), interatrial septum (IAS), LA, left atrial appendage (LAA) and PVs. Having checked the overall structure of atria and ventricles, as well as the presence of pericardial effusion (PE) and thrombi, ICE was advanced and rotated past the aortic valve to visualize FO. The TS needle was advanced to RA through the Swartz sheath. Under ICE guidance to choose an optimal TS position, orienting toward the LPV, the Swartz sheath was advanced to tent the FO. The TS needle was advanced until a “pop” was felt and visualized as it crossed the IAS. A small amount of saline was then injected to create microbubbles in the LA and to confirm successful LA access. The sheath was advanced over the needle, then the needle was withdrawn and the ablation catheter was advanced. For those who underwent left atrial appendage occlusion (LAAO) following RFCA, a hard guide wire was first advanced to the LA to perform LAA angiography. The occlusion device, with size and shape selected according to the LAA anatomy, was then delivered to the LAA through the delivery sheath. After withdrawal of the delivery sheath, the occlusion device was released and carefully positioned, ensuring no contrast agent leakage between the device and the LAA on angiography. Procedural duration and post-procedural test results were collected from the HIS.

**Figure 1:**
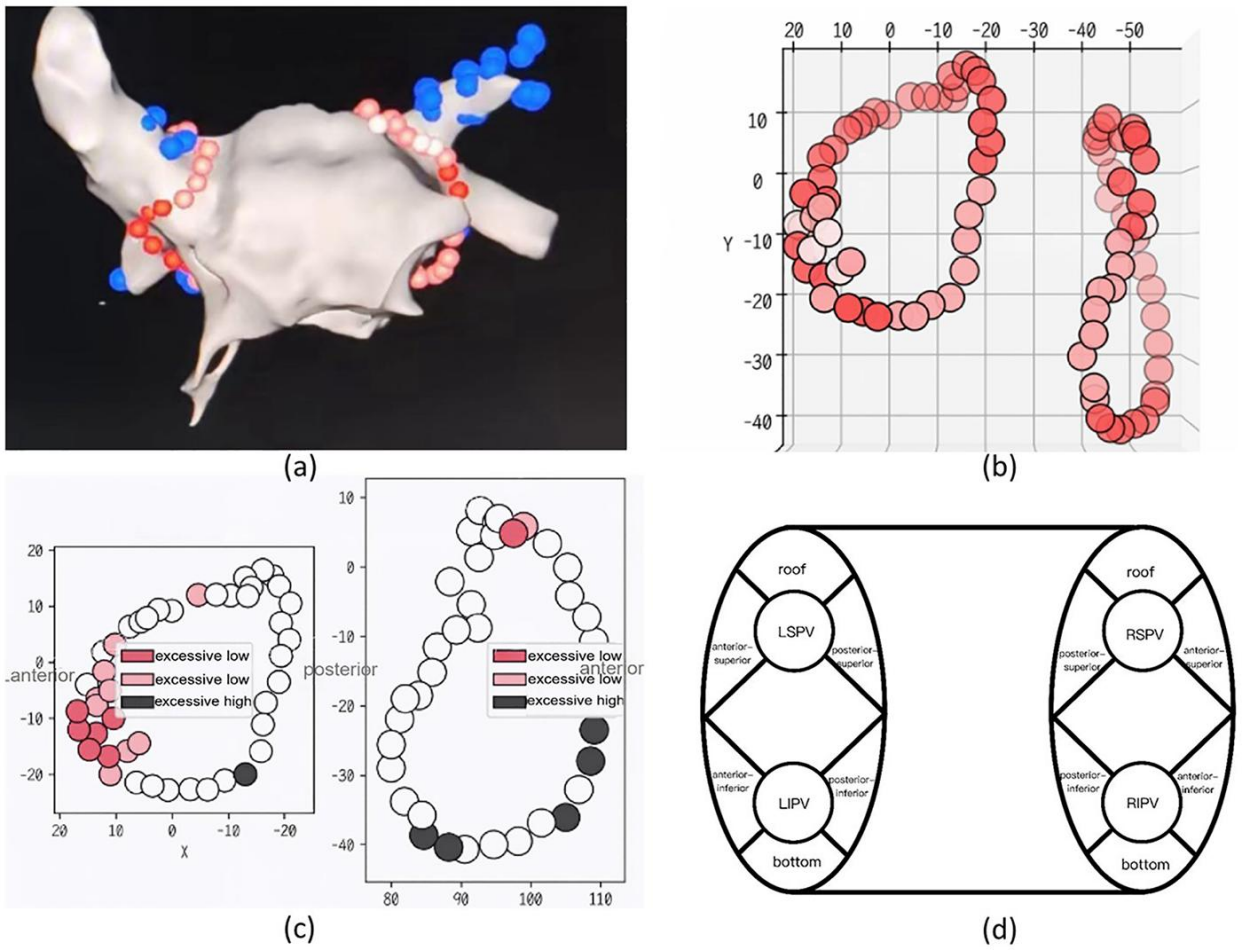
The segments of bilateral pulmonary vein antrum (PVA). **(a)** The ablation lesions visualized via Vizigo system. **(b)** Visualization of ablation lesions that isolate bilateral PVAs. **(c)** In AIFV system, ablation lesions with excessive low FOT are presented in red, while with excessive high FOT in black. **(d)** Bilateral PVAs were divided into 12 segments. LF, left roof; LAS, left anterior-superior; LAI, left anterior-inferior; LPS, left posterior-superior; LPI, left posterior-inferior; LB, left bottom; LSPV, left superior pulmonary vein; LIPV, left inferior pulmonary vein; RSPV, right superior pulmonary vein; RIPV, right inferior pulmonary vein.

As illustrated in Figure 1, the bilateral PVA and ablation circles were divided into 12 segments. The left roof (LF) segment is located superior to the left superior pulmonary vein (LSPV) on the roof of LA. The left anterior-superior (LAS) segment is situated anterolaterally to the LSPV. The left anterior-inferior (LAI) segment is located anterolaterally to the left inferior pulmonary vein (LIPV). The left posterior-superior (LPS) segment is adjacent to the posterior aspect of the LSPV on the posterior wall of the LA. The left posterior-inferior (LPI) segment is adjacent to the posterior aspect of the LIPV. The left bottom (LB) segment is adjacent to the inferior aspect of the LIPV on the posterior wall of the LA. The right roof (RF) segment is located superior to the right superior pulmonary vein (RSPV) on the roof of the LA. The right anterior-superior (RAS) segment is situated anterolaterally to the RSPV. The right anterior-inferior (RAI) segment is located anterolaterally to the right inferior pulmonary vein (RIPV). The right posterior-superior (RPS) segment is adjacent to the posterior aspect of the RSPV on the posterior wall of the LA. The right posterior-inferior (RPI) segment is adjacent to the posterior aspect of the RIPV. The right bottom (RB) segment is adjacent to the inferior aspect of the RIPV on the posterior wall of the LA. Force-over-time (FOT) is defined as the proportion of time during which the catheter contact force (CF) at an ablation lesion meets a preset value relative to the total ablation time for that lesion. Appropriate FOT was preset as the proportion of time during which the CF is within the range of 5–15 g accounting for 40%–60% of the total ablation time. The number of ablation lesions with FOT below this standard were defined as count with low FOT, while the number of those above this standard were defined as count with high FOT. FOT were calculated from the Vizigo system. Ablation gaps in the PV circuits were defined as distances between adjacent lesions exceeding 8 mm. Gap counts were recorded for every ablation circles. FPI was categorized as PVs without gaps. The raw data of ablation were collected from Carto 3 system and further analyzed by Johnson & Johnson's BW AIFV intelligent analysis system. The ablation-related data, including ablation duration, gap counts and locations, and FOT conditions, were collected from AIFV analysis system.

### 2.3 Follow-up

Medical records and test results were collected from HIS. The first three months following RFCA were defined as the blanking period (BP), during which the incidence of atrial tachyarrhythmias (ATA) was analyzed and defined as early recurrence (ER). Recurrence was defined as any episode of ATA lasting longer than 30 s and documented by both 12-lead routine ECG and 24 h Holter monitoring after the BP. Cardiac structural and functional changes were evaluated using echocardiography. For patients scheduled for routine follow-up, telephone follow-up was conducted using contact information recorded in the HIS.

### 2.4 Statistical analysis

Normality of continuous variables was assessed using Shapiro–Wilk test, while homogeneity of variance for normally distributed variables was evaluated via Levene's test. Continuous variables meeting both assumptions were presented as mean ± standard deviation (x ± s) and compared between groups using Student's *t*-test. Variables not meeting these assumptions were described using median and interquartile range and compared with the Mann–Whitney *U*-test. Categorical variables were presented as counts and percentages (%) and compared using the Chi-square test or Fisher's exact test, depending on expected frequencies. Propensity score matching (PSM) was performed using the nearest neighbor method without replacement, with a matching ratio of 1:1. The standardized mean differences (SMD) for all adjusted variables were below 0.02 after propensity score matching. Kendall's correlation test was employed to identify continuous variables significantly associated with procedure duration, while the point-biserial correlation coefficient was used for binary variables. Variables identified through these methods, along with those previously reported to influence procedure duration, were included in a multivariate Gamma regression model to analyze factors affecting procedure duration. A similar approach was applied to analyze factors influencing ablation time. The proportional hazards assumption (PHA) was verified using the Schoenfeld test. Univariate Cox regression was performed to screen factors influencing recurrence, and the selected variables were included in a multivariate Cox regression analysis. Using ICE application as the grouping variable, Kaplan–Meier survival analysis was conducted, and the log-rank test was used for comparison to investigate whether there was a difference in postoperative recurrence between the two groups. All statistical analyses were conducted in R Studio (version 4.5.1).

## 3 Results

### 3.1 Characteristics before PSM

#### 3.1.1 Baseline characteristics

A total of 167 patients undergoing first-time RFCA for AF at the Shinan Campus of the Affiliated Hospital of Qingdao University between June 2022 and June 2023 were included. ICE guidance was used in 84 patients, while 83 patients underwent the procedure without ICE. The mean ages in the ICE and non-ICE groups were 62.58 ± 8.66 years and 61.69 ± 9.75 years, respectively (*P* = 0.535), and male patients accounted for 60.2% and 60.7%, respectively (*P* = 0.950). The mean body mass index (BMI) was 26.15 ± 2.47 kg/m² in the ICE group and 25.60 ± 2.98 kg/m² in the non-ICE group, with no statistically significant difference between the groups (*P* = 0.198).

Prior to PSM, statistically significant differences were observed between the two groups regarding AF type and certain comorbidities. PAF accounted for 85.5% in ICE group and 70.2% in non-ICE group (*P* = 0.017). LVEF (60 vs. 58, *P* = 0.022) and estimated glomerular filtration rate (eGFR, 107.24 vs. 92.52, *P* < 0.001) was higher in ICE group, while creatine (Cr, 55.00 vs. 68.00, *P* = 0.005) was lower. There was a higher prevalence of coronary artery disease (CAD, 43.4% vs. 25.0%, *P* = 0.012), and a lower prevalence of cerebrovascular events (CVE, 6.0% vs. 32.1%, *P* < 0.001) in the ICE group (Table 1). Other baseline characteristics were comparable between the groups.

**Table 1:** Baseline characteristic before and after propensity score matching (PSM).

Variable	Pre-PSM			Post-PSM		
	ICE (*n* = 84)	Non-ICE (*n* = 83)	*P* value	ICE (*n* = 51)	Non-ICE (*n* = 51)	*P* value
Age (year, Q1–Q3)	62.58 ± 8.66	61.69 ± 9.75	0.535^a^	62.00 (55.50–0.50)	61.00 (57.00–66.50)	0.656^b^
Gender [male (%)]	50 (60.2%)	51 (60.7%)	0.950^c^	29 (56.9%)	28 (54.9%)	0.842^c^
BMI (Kg/m^2^, x ± s)	26.15 ± 2.47	25.60 ± 2.98	0.198^a^	25.81 ± 2.95	25.85 ± 2.57	0.955^a^
AF type (%)			0.017*^,^^c^			1.000^c^
PAF	71 (85.5%)	59 (70.2%)		41 (80.4%)	41 (80.4%)	
PeAF	12 (14.5%)	25 (29.8%)		10 (19.6%)	10 (19.6%)	
AF duration (month, Q1–Q3)	24.00 (4.00,60.00)	24.00 (6.00,60.00)	0.427^b^	24.00 (6.00–72.00)	12.00 (6.00–48.00)	0.394^b^
Smoking (%)	14 (16.9%)	16 (19.0%)	0.714^c^	8 (15.7%)	7 (13.7%)	0.780^c^
Alcohol consumption (%)	13 (15.7%)	15 (17.9%)	0.704^c^	9 (17.6%)	7 (13.7%)	0.586^c^
MR (%)	3 (3.6%)	5 (6.0%)	0.720^d^	1 (2.0%)	3 (5.9%)	0.610^d^
VHD (%)	3 (3.6%)	7 (8.3%)	0.329^d^	2 (3.9%)	3 (5.9%)	1.000^d^
LAD (mm, Q1–Q3)	40.17 ± 5.76	41.74 ± 5.73	0.079^a^	40.78 ± 5.53	39.73 ± 6.28	0.368^a^
LVEF (%, Q1–Q3)	60 (55–61)	58 (55–60)	0.022^b^	60 (55–60)	60 (55–61)	0.754^b^
HF (%)	10 (12.0%)	13 (15.5%)	0.520^c^	6 (11.8%)	6 (11.8%)	1.000^c^
Hypertension (%)	46 (55.4%)	43 (51.2%)	0.584^c^	30 (58.8%)	27 (52.9%)	0.550^c^
Cardiomyopathy (%)	1 (1.2%)	3 (3.6%)	0.620^e^	1 (2.0%)	1 (2.0%)	1.000^d^
CAD (%)	36 (43.4%)	21 (25.0%)	0.012*^,^^c^	17 (33.3%)	16 (31.4%)	0.832^c^
MI (%)	1 (1.2%)	0 (0.0%)	0.497^e^	0 (0.0%)	0 (0.0%)	1.000^c^
CVE (%)	5 (6.0%)	27 (32.1%)	<0.001***^,^^c^	6 (11.8%)	5 (9.8%)	0.750^c^
PVD (%)	3 (3.6%)	6 (7.1%)	0.496^d^	4 (7.8%)	3 (5.9%)	1.000^d^
Diabetes (%)	19 (22.9%)	21 (25.0%)	0.750^c^	12 (23.5%)	11 (21.6%)	0.813^c^
Hyperlipidemia (%)	63 (75.9%)	69 (82.1%)	0.322^c^	40 (78.4%)	38 (74.5%)	0.641^c^
Thyroid disease (%)	24 (28.9%)	25 (30.1%)	0.865^c^	15 (29.4%)	14 (27.5%)	0.826^c^
Cr (μmol/L, Q1–Q3)	69.00 (62.00–82.00)	66.50 (56.50–75.50)	0.245^b^	66.00 (55.00–75.50)	55.00 (51.00–79.00)	0.313^b^
eGFR [ml/(min*1.73m^2^), Q1–Q3]	90.62 (82.50–99.10)	93.68 (86.27–97.19)	0.699^b^	92.93 (78.76–101.75)	96.96 (73.00–107.79)	0.290^b^
CHA_2_DS_2_-VASc score (%)			0.562^e^			0.866^e^
0	10 (12.0%)	6 (7.1%)		5 (9.8%)	7 (13.7%)	
1	17 (20.5%)	16 (19.0%)		8 (15.7%)	11 (21.6%)	
2	19 (22.9%)	17 (20.2%)		14 (27.5%)	11 (21.6%)	
3	24 (28.9%)	19 (22.6%)		11 (21.6%)	13 (25.5%)	
4	7 (8.4%)	13 (15.5%)		7 (13.7%)	5 (9.8%)	
5	3 (3.6%)	6 (7.1%)		4 (7.8%)	2 (3.9%)	
6	2 (2.4%)	5 (6.0%)		1 (2.0%)	2 (3.9%)	
7	1 (1.2%)	1 (1.2%)		0 (0.0%)	0 (0.0%)	
8	0 (0.0%)	1 (1.2%)		1 (2.0%)	0 (0.0%)	

ICE, intracardiac echocardiography; BMI, body mass index; AF, atrial fibrillation; PAF, paroxysmal AF; PeAF, persistent AF; MR, mitral regurgitation; VHD, valvular heart disease; LAD, left atrium diameter; LVEF, left ventricular ejection fraction; HF, heart failure; CAD, coronary artery disease; MI, myocardial infarction; CVE, cerebrovascular events; PVD, peripheral vascular disease; Cr, creatinine; eGFR, estimated glomerular filtration rate.

a Student's *t*-test.

b Mann–Whitney *U*-test.

c *χ*^2^ test.

d Yate's correct test.

e Fisher's exact test.

*P-value <0.05, ***P-value <0.001.

#### 3.1.2 Procedural metrics

Prior to PSM, a statistically significant difference was observed between the two groups regarding LAAO. The proportion of patients who underwent LAAO was 28.9% in the ICE group compared to 48.8% in the non-ICE group (*P* = 0.008) (Table 2).

**Table 2:** Procedural characteristics and ablation-related characteristics.

Variable	Pre-PSM			Post-PSM		
	ICE (*n* = 84)	Non-ICE (*n* = 83)	*P* value	ICE (*n* = 51)	Non-ICE (*n* = 51)	*P* value
LAAO (%)	24 (28.9%)	41 (48.8%)	0.008*	20 (39.2%)	18 (35.3%)	0.682^b^
Procedure duration (minute, Q1–Q3)	60.0 (50.0–130.0)	77.5 (59.0–105.5)	0.091^a^	75.0 (59.0–100.0)	50.0 (50.0–115.0)	0.041*^,^^a^
Ablation duration (minute, Q1–Q3)	42.0 (23.0–63.0)	36.0 (30.0–61.5)	0.761^a^	36.0 (29.0–54.0)	32.0 (22.0–63.0)	0.524^a^
LGC (Q1–Q3)	0 (0–1)	0 (0–1)	0.314^a^	0 (0–1)	0 (0–1)	0.917^a^
RGC (Q1–Q3)	0 (0–2)	0 (0–1)	0.053^a^	0 (0–2)	1 (0–2)	0.092^a^
LFPI (%)	57 (68.7%)	46 (54.8%)	0.064^b^	29 (56.7%)	35 (68.6%)	0.219^b^
RFPI (%)	46 (55.4%)	57 (67.9%)	0.098^b^	34 (66.7%)	27 (52.9%)	0.157^b^
TFPI (%)	36 (43.4%)	36 (42.9%)	0.946^b^	21 (41.2%)	17 (33.3%)	0.413^b^
Follow-up (month, Q1–Q3)	17.0 (14.0–19.0)	17.5 (13.5–19.5)	0.280^a^	17.0 (13.0–19.0)	17.0 (14.0–19.0)	0.971^a^
Complication (%)	0 (0.0%)	4 (4.8%)	0.121^c^	2 (3.9%)	0 (0.0%)	0.475^c^
ER (%)	5 (6.0%)	10 (11.9%)	0.184^b^	6 (11.8%)	4 (7.8%)	0.505^b^
Recurrence (%)	33 (39.8%)	27 (32.1%)	0.305^b^	15 (29.4%)	21 (41.2%)	0.214^b^

LAAO, left atrial appendage occlusion; FOT, force over time; LGC, gap count of left pulmonary vein antrum; RGC, gap count of right pulmonary vein antrum; LFPI, first-pass isolation of left pulmonary vein antrum; RFPI, first-pass isolation of right pulmonary vein antrum; TFPI, first-pass isolation of bilateral pulmonary vein antra; ER, early recurrence.

a Mann–Whitney *U*-test.

b *χ*^2^ test.

c Fisher's exact test.

*P-value < 0.05.

#### 3.1.3 Distribution of lesions with abnormal FOT

Before PSM, the distributions of abnormal FOT lesion counts differed significantly between the ICE group and non-ICE group in several PV segments, particularly those adjacent to the right pulmonary veins. Near the right pulmonary veins, significant differences were observed in the low FOT count in RAS segment [0 (0–5) vs. 0 (0–1), *P* = 0.021], low FOT count in RPI segment [3 (0–4) vs. 0 (0–3), *P* = 0.006], low FOT count and high FOT count in RB segment [0 (0–2) vs. 0 (0–1), *P* = 0.002; 5 (2–6) vs. 3 (1–5), *P* = 0.006], high FOT count in RF segment [0 (0–2) vs. 0 (0–1), *P* = 0.042], high FOT count in RAS segment [6 (1–6) vs. 2 (1–6), *P* = 0.006], high FOT count in RAI segment [7 (3–7) vs. 4 (2–7), *P* = 0.017], and high FOT count in RPS segment [4 (0–4) vs. 1 (0–4), *P* = 0.004]. Near the left pulmonary veins, significant differences were observed in the low FOT count in LPS segment [0 (0–1) vs. 0 (0–1), *P* = 0.048], high FOT count in LAS segment [0 (0–1) vs. 0 (0–0), *P* = 0.031], high FOT count in LAI segment [0 (0–1) vs. 0 (0–0), *P* = 0.027], and high FOT count in LPI segment [0 (0–1) vs. 0 (0–1), *P* = 0.035]. Before PSM, the remaining PV segments showed no significant differences in abnormal FOT lesion counts between the two groups (Table 3).

**Table 3:** Distribution of ablation lesions with abnormal FOT.

Number of lesions with abnormal FOT	Pre-PSM			Post-PSM		
	ICE (*n* = 84)	Non-ICE (*n* = 83)	*P* value^a^	ICE (*n* = 51)	Non-ICE (*n* = 51)	*P* value^a^
Low FOT on left circle (Q1–Q3)						
LF segment	1 (0–4)	1 (0–3)	0.187	0 (0–3)	0 (0–4)	0.205
LAS segment	5 (0–9)	4 (2–9)	0.740	4 (3–8)	2 (0–9)	0.23
LAI segment	10 (2–11)	6 (2–10)	0.075	5 (1–9)	10 (2–11)	0.051
LPS segment	0 (0–1)	0 (0–1)	0.048*	0 (0–1)	1 (0–1)	0.013*
LPI segment	0 (0–1)	0 (0–0)	0.086	0 (0–0)	0 (0–2)	0.007**
LB segment	0 (0–0)	0 (0–1)	0.955	0 (0–0)	0 (0–0)	0.773
High FOT on left circle (Q1–Q3)						
LF segment	0 (0–1)	0 (0–1)	0.406	0 (0–1)	0 (0–2)	0.99
LAS segment	0 (0–1)	0 (0–0)	0.031*	0 (0–0)	0 (0–1)	0.071
LAI segment	0 (0–1)	0 (0–0)	0.027*	0 (0–0)	0 (0–1)	0.255
LPS segment	0 (0–3)	0 (0–2)	0.361	0 (0–1)	1 (0–4)	0.019*
LPI segment	0 (0–1)	0 (0–1)	0.035*	0 (0–0)	0 (0–1)	0.029*
LB segment	0 (0–0)	0 (0–0)	0.456	0 (0–0)	0 (0–0)	0.985
Low FOT on right circle (Q1–Q3)						
RF segment	3 (0–5)	2 (0–5)	0.373	2 (0–5)	3 (0–5)	0.701
RAS segment	0 (0–5)	0 (0–1)	0.021*	0 (0–1)	2 (0–5)	0.001**
RAI segment	0 (0–0)	0 (0–0)	0.221	0 (0–0)	0 (0–0)	0.777
RPS segment	2 (0–3)	1 (0–2)	0.025*	1 (0–2)	1 (0–3)	0.175
RPI segment	3 (0–4)	0 (0–3)	0.006**	0 (0–3)	2 (0–4)	0.203
RB segment	0 (0–2)	0 (0–1)	0.002**	0 (0–1)	0 (0–2)	0.017*
High FOT on right circle (Q1–Q3)						
RF segment	0 (0–2)	0 (0–1)	0.042*	0 (0–1)	0 (0–2)	0.157
RAS segment	6 (1–6)	2 (1–6)	0.006**	2 (1–4)	6 (1–6)	0.088
RAI segment	7 (3–7)	4 (2–7)	0.017*	4 (2–6)	7 (2–7)	0.246
RPS segment	4 (0–4)	1 (0–4)	0.004**	1 (0–4)	4 (1–4)	0.014*
RPI segment	0 (0–2)	0 (0–1)	0.202	0 (0–1)	0 (0–3)	0.185
RB segment	5 (2–6)	3 (1–5)	0.006**	3 (1–5)	5 (2–6)	0.091

LF, left roof; LAS, left anterior-superior; LAI, left anterior-inferior; LPS, left posterior-superior; LPI, left posterior-inferior; LB, left bottom.

a Mann–Whitney *U*-test.

*P-value <0.05, **P-value <0.01.

### 3.2 Characteristics after PSM

#### 3.2.1 Baseline characteristics

**Figure 2:**
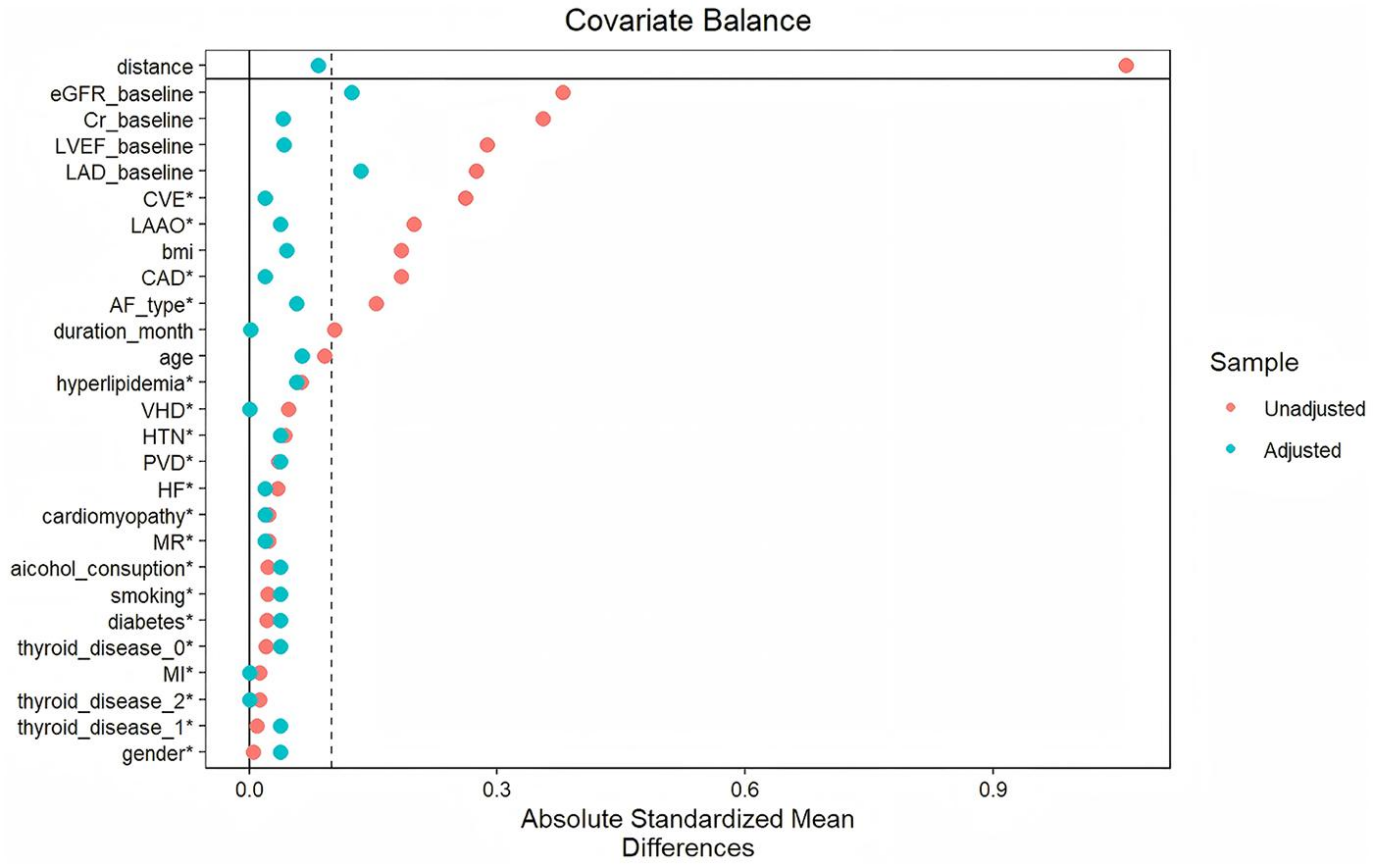
Pre-PSM and post-PSM standardized mean differences (SMD) of covariates.

For PSM, 1:1 nearest-neighbor matching without replacement was performed. The propensity score was estimated using logistic regression with ICE as the exposure variable. A caliper width of 0.2 was applie. The following covariates were included in the propensity model: age, BMI, AF type, AF duration, smoking, alcohol consumption, LAD, LVEF, eGFR, Cr, CAD, CVE, hypertension, cardiomyopathy, myocardial infarction (MI), heart failure (HF), valvular heart disease, peripheral vascular disease, hyperlipidemia, diabetes, history of thyroid disease and LAAO during the procedure. Covariate balance before and after matching was assessed using SMD. As shown in Figure 2, after PSM the absolute SMDs for all covariates were <0.2, and SMDs for variables with the greatest pre-match imbalance were substantially reduced, indicating that matching improved between-group comparability. After completing PSM, 102 patients were retained for subsequent analyses, comprising 51 patients in the ICE group and 51 patients in the non-ICE group (Figure 2).

After PSM, no statistically significant differences were observed in the baseline data between the two groups (Table 1).

#### 3.2.2 Procedural metrics

After PSM, the proportion of patients who underwent LAAO was 39.2% in the ICE group and 35.5% in the non-ICE group, with no statistically significant difference between the two groups (*P* = 0.682). The ablation time was [36.0 (29.0–54.0)] min in the ICE group and [32.0 (22.0–63.0)] min in the non-ICE group, showing no statistically significant difference (*P* = 0.524). However, a significant statistical difference was observed in the total procedure time between the two groups. The procedure time was [75.0 (59.0–100.0)] min in the ICE group compared to [50.0 (50.0–115.0)] min in the non-ICE group (*P* = 0.041) (Table 2).

Regarding the achievement of first-pass isolation (FPI) after PSM, there were no statistically significant differences between the two groups in LFPI (56.7% vs. 68.6%, *P* = 0.219), RFPI (66.7% vs. 52.9%, *P* = 0.157), or TFPI (41.2% vs. 33.3%, *P* = 0.413).

#### 3.2.3 Distribution of lesions with abnormal FOT

After PSM, the distributions of abnormal FOT lesion counts remained significantly different between the two groups in several PV segments. Near the left pulmonary veins, significant differences were observed in the low and high FOT count in LPS segment [0 (0–1) vs. 1 (0–1), *P* = 0.013; 0 (0–1) vs. 1 (0–4), *P* = 0.019] and the low and high FOT count in LPI segment [l0 (0–0) vs. 0 (0–2), *P* = 0.007; 0 (0–0) vs. 0 (0–1), *P* = 0.029]. Near the right pulmonary veins, significant differences were observed in the low FOT count in RAS segment [0 (0–1) vs. 2 (0–5), *P* = 0.001], low FOT count in RB segment [0 (0–1) vs. 0 (0–2), *P* = 0.017], and high FOT count in RPS segment [1 (0–4) vs. 4 (1–4), *P* = 0.014]. After PSM, the remaining PV antral subsegments showed no significant between-group differences in abnormal FOT lesion counts (Table 3).

### 3.3 Recurrence predictors

To investigate the factors influencing recurrence, this study first incorporated baseline variables and procedure-related variables into univariate Cox regression analysis. The variables with a *P*-value <0.10 were selected and included in a multivariate Cox regression analysis, with a *P*-value <0.05 used to identify independent factors associated with recurrence. The results are detailed in Table 4. The variables the high FOT count in RAS segment (ZPH-*P* = 0.04) and ER (ZPH-*P* = 0.03) did not meet the PHA and were excluded from subsequent analyses.

**Table 4:** Univariate and multivariate Cox regression of recurrence.

Characteristics	Univariate Cox			Multivariate Cox		
	Beta	HR (95% CI)	*P* value	Beta	HR (95% CI)	*P* value
eGFR	−0.02	0.98 (0.96–1.00)	0.012*	−0.03	0.97 (0.94–0.99)	0.017*
High FOT count in RPS segment	0.33	1.40 (1.14–1.71)	0.001**	0.39	1.48 (1.05–2.09)	0.025*
Low FOT count in LPS segment	0.45	1.57 (1.21–2.05)	<0.001***			
RGC	0.30	1.35 (1.13–1.62)	0.001**			
Low FOT count in LAI segment	0.12	1.13 (1.04–1.23)	0.003**			
Low FOT count in LPI segment	0.33	1.40 (1.11–1.75)	0.004**			
PVD	1.38	3.97 (1.5–10.54)	0.006**			
Low FOT count in RAS segment	0.18	1.20 (1.05–1.38)	0.009**			
High FOT count in RAS segment	0.13	1.14 (1.02–1.29)	0.025*			
LAAO	0.80	2.23 (1.10–4.52)	0.026*			
ICE	−0.82	0.44 (0.21–0.93)	0.032*			
High FOT count in LPS segment	0.21	1.24 (1.01–1.51)	0.036*			
High FOT count in RPI segment	0.20	1.23 (1.01–1.48)	0.036*			

*P-value <0.05, **P-value <0.01, ***P-value <0.001.

**Figure 3:**
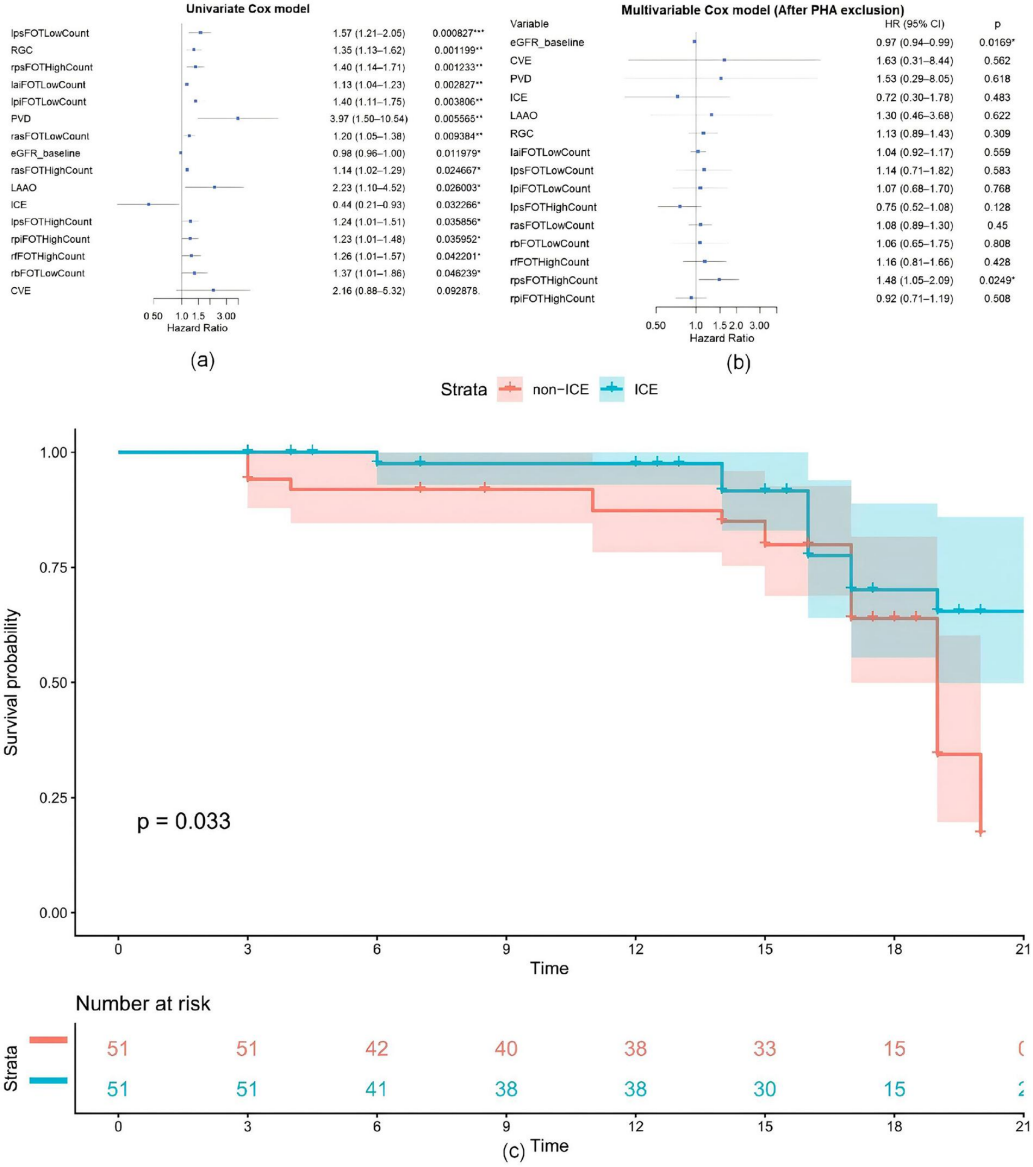
Predictors of recurrence. **(a)** Univariate Cox regression forestplot. **(b)** Multivariate Cox regression forestplot. **(c)** Kaplan–Meier survival curve with ICE use as grouping factor.

As shown in Figure 3, in univariable Cox regression analysis, baseline eGFR (HR = 0.98, 95% CI 0.96–1.00, *P* = 0.012) and ICE (HR = 0.44, 95% CI 0.21–0.93, *P* = 0.032) were associated with a lower risk of recurrence. By contrast, the high FOT count in RPS segment (HR = 1.40, 95% CI 1.14–1.71, *P* = 0.001), the low FOT count in LPS segment (HR = 1.57, 95% CI 1.21–2.05, *P* < 0.001), RGC (HR = 1.35, 95% CI 1.13–1.62, *P* = 0.001), the low FOT count in LAI segment (HR = 1.13, 95% CI 1.04–1.23, *P* = 0.003), the low FOT count in LPI segment (HR = 1.40, 95% CI 1.11–1.75, *P* = 0.004), PVD (HR = 3.97, 95% CI 1.50–10.54, *P* = 0.006), the low FOT count in RAS segment (HR = 1.20, 95% CI 1.05–1.38, *P* = 0.009), the high FOT count in RAS segment (HR = 1.14, 95% CI 1.02–1.29, *P* = 0.025), LAAO (HR = 2.23, 95% CI 1.10–4.52, *P* = 0.026), the high FOT count in LPS segment (HR = 1.24, 95% CI 1.01–1.51, *P* = 0.036), the high FOT count in RPI segment (HR = 1.23, 95% CI 1.01–1.48, *P* = 0.036), the high FOT count in RF segment (HR = 1.26, 95% CI 1.01–1.57, *P* = 0.042), the low FOT count in RB segment (HR = 1.37, 95% CI 1.01–1.86, *P* = 0.046), and CVE (HR = 2.16, 95% CI 0.88–5.32, *P* = 0.093) were associated with a higher risk of recurrence. The variables above were included in multivariable Cox regression analysis. After adjusting for confounders, baseline eGFR (HR = 0.97, 95% CI 0.94–0.99, *P* = 0.017) and the high FOT count in RPS segment (HR = 1.48, 95% CI 1.05–2.09, *P* = 0.025) remained independent predictors of recurrence. Higher baseline eGFR was identified as an independent protective factor for maintaining sinus rhythm, whereas an increased the high FOT count in RPS segment was found to be an independent risk factor for recurrence.

In the Kaplan–Meier analysis, a statistically significant difference in recurrence was observed between the ICE group and the non-ICE group (*P* = 0.033).

### 3.4 Factors affecting ablation duration

**Figure 4:**
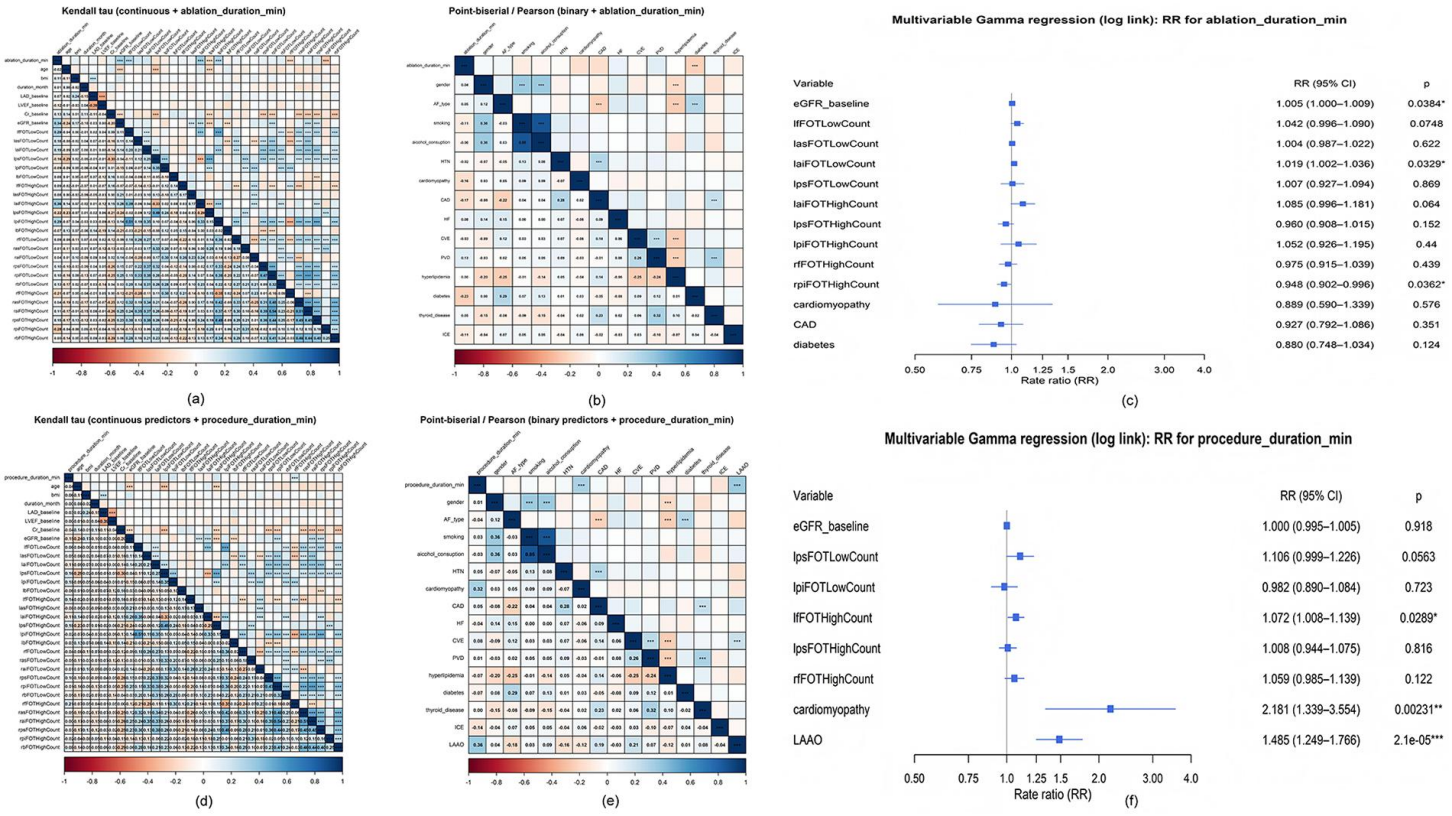
Heatmap of kendall correlation test results of ablation duration and procedural duration with continuous variables and forest plots of the gamma analysis. **(a)** Kendall correlation test results of ablation duration and the relative factors including baseline characteristics and procedural data. **(b)** Point-biserial correlation test results of ablation duration and the relative factors including baseline characteristics and procedural data. **(c)** Forest plot of Gamma analysis results of independent factors of ablation duration. **(d)** Kendall correlation test results of procedural duration and the relative factors including baseline characteristics and procedural data. **(e)** Point-biserial correlation test results of procedural duration and the relative factors including baseline characteristics and procedural data. **(f)** Forest plot of Gamma analysis results of independent factors of procedure duration.

To investigate factors associated with ablation time, Kendall's correlation test was employed for continuous variables, with the corresponding correlation heatmap presented in Figure 4. For dichotomous variables, the point-biserial correlation analysis was conducted, and its results are visualized in the correlation heatmap shown in Figure 4. The Kendall correlation test revealed that several variables were positively correlated with ablation time. These included the high FOT count in LAI segment (*τ* = 0.365, *P* < 0.001), baseline eGFR (*τ* = 0.337, *P* < 0.001), the low FOT count in LF segment (*τ* = 0.293, *P* = 0.002), the high FOT count in LPI segment (*τ* = 0.292, *P* = 0.002), the low FOT count in LAI segment (*τ* = 0.190, *P* = 0.051), and the low FOT count in LAS segment (*τ* = 0.183, *P* = 0.061). Conversely, several variables showed a negative correlation with ablation time. These were the high FOT count in RPI segment (*τ* = −0.276, *P* = 0.004), the high FOT count in LPS segment (*τ* = −0.218, *P* = 0.024), the high FOT count in RF segment (*τ* = −0.211, *P* = 0.030), and the low FOT count in LPS segment (*τ* = −0.164, *P* = 0.093). The point-biserial correlation test revealed that diabetes (*r* = −0.226, *P* = 0.020), CAD (*r* = −0.171, *P* = 0.079), and cardiomyopathy (*r* = −0.163, *P* = 0.094) were negatively correlated with ablation time.

The selected variables were incorporated into a multivariate Gamma regression analysis. After adjusting for confounding factors, the results showed that baseline eGFR (RR = 1.005, 95% CI 1.000–1.009, *P* = 0.038) and the low FOT count in LAI segment (RR = 1.019, 95% CI 1.002–1.036, *P* = 0.033) were factors associated with prolonged ablation time. Conversely, the high FOT count in RPI segment (RR = 0.948, 95% CI 0.902–0.996, *P* = 0.036) was the factor associated with shortened ablation time (Figure 4).

### 3.5 Factors affecting procedural duration

To investigate factors associated with procedural duration time, Kendall's correlation test was employed for continuous variables. For dichotomous variables, the point-biserial correlation analysis was conducted. According to Kendall's correlation analysis, procedural duration was positively correlated with the high FOT count in RF segment (*τ* = 0.206, *P* = 0.034), the low FOT count in LPI segment (*τ* = 0.185, *P* = 0.058), the high FOT count in LPS segment (*τ* = 0.183, *P* = 0.061), and the low FOT count in LPS segment (*τ* = 0.163, *P* = 0.097). The high FOT count in LF segment (*τ* = 0.142, *P* = 0.146) was also positively correlated with operative time, but this association was not statistically significant. The eGFR (*τ* = −0.146, *P* = 0.135) was negatively correlated with operative time, likewise without statistical significance. Point-biserial correlation analysis showed that LAAO (*r* = 0.359, *P* < 0.001) and cardiomyopathy (*r* = 0.318, *P* < 0.001) were positively correlated with procedural duration.

Variables identified in the correlation analysis were entered into a multivariable Gamma regression model. After adjustment for confounders, high FOT count in LF segment (RR = 1.072, 95% CI 1.008–1.139, *P* = 0.029), cardiomyopathy (RR = 2.181, 95% CI 1.339–3.554, *P* = 0.002), and LAAO (RR = 1.485, 95% CI 1.249–1.766, *P* < 0.001) were independently associated with longer procedural duration.

**Figure 5:**
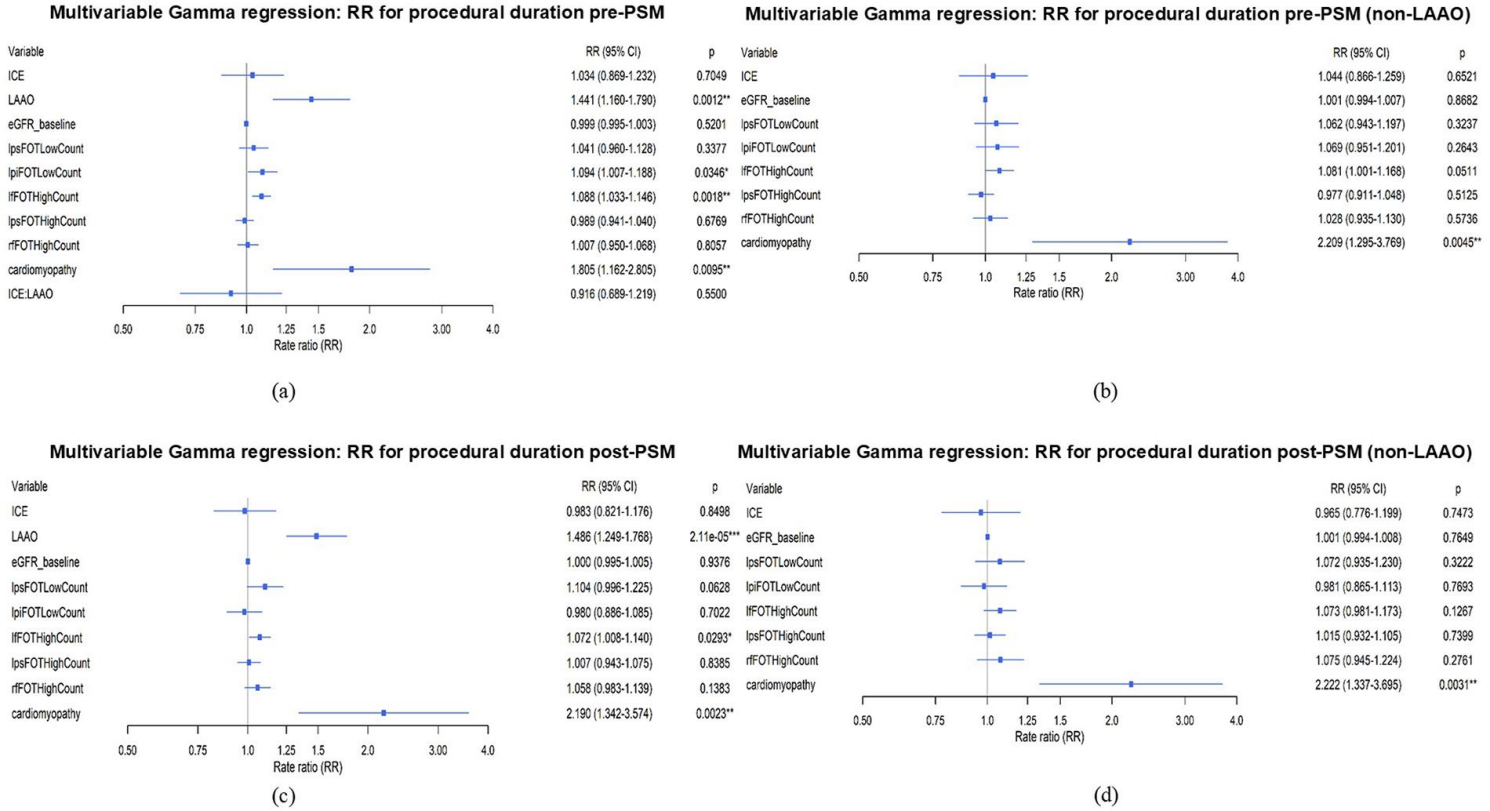
Subgroup analysis. **(a)** Multivariable gamma regression in all cases before PSM. **(b)** Multivariable gamma regression in cases excluding LAAO before PSM. **(c)** Multivariable gamma regression in all cases after PSM. **(b)** Multivariable gamma regression in cases excluding LAAO after PSM.

To further minimize potential confounding from LAAO, a subgroup analysis was performed (Figure 5). In multivariable Gamma regression in the full cohort, ICE was not associated with longer procedure duration (RR = 1.034, 95% CI 0.869–1.232, *P* = 0.705). LAAO was significantly associated with longer duration (RR = 1.441, 95% CI 1.160–1.790, *P* = 0.001). After adding the interaction term, the ICE × LAAO effect was not significant (*P* = 0.550). To further minimize potential confounding from LAAO, we performed a restricted analysis excluding LAAO cases, in which ICE remained not associated with longer procedure duration (RR = 1.044, 95% CI 0.866–1.259, *P* = 0.652). Findings were consistent in the PSM cohort. ICE was not associated with longer procedure duration (RR = 0.983, 95% CI 0.821–1.176, *P* = 0.850). LAAO was significantly associated with longer duration (RR = 1.486, 95% CI 1.249–1.768, *P* < 0.001). In post-PSM subgroup excluding LAAO cases, ICE was not associated with longer procedure duration (RR = 0.965, 95% CI 0.776–1.199, *P* = 0.747). Collectively, these results suggest that the impact of ICE on RFCA procedural duration is not primarily driven by concomitant LAAO.

## 4 Discussion

This study found that ICE influenced abnormal FOT in some PVA segments, procedural and ablation duration. Abnormal FOT in some segments were linked to increased AF recurrence, whereas higher eGFR reduced the risk of recurrence. ICE-guided procedures demonstrated decrease in AF recurrence, while the benefit may not be directly provided by ICE itself but rather mediated through the improvements of certain procedural characteristics.

Baseline imbalances were present among patients included in this study. After PSM, intergroup heterogeneity was improved. In the field of catheter ablation for AF, procedural outcomes differ substantially across patient subgroups with different clinical characteristics. Previous studies have identified AF type as a strong predictor of AF recurrence. Age, gender and diagnosis-to-ablation time (DAT) are independent predictors (23–27). Regarding lifestyle, smoking and alcohol consumption are associated with new-onset AF (28). Comorbidities such as hypertension, CAD, cardiomyopathy, valvular heart disease, diabetes, peripheral vascular disease and thyroid disorders are all risk factors for AF (6, 29–32). Further researches on comorbidities and cardiovascular risk factors indicates that obesity, HF and higher CHA2DS2-VASc scores are risk factors for recurrence after CA (33–35). Chronic kidney disease (CKD) may increase recurrence risk, and the uric acid/albumin ratio (UAR) is a predictor in non-diabetic patients (2, 36, 37). The AURORA study found early recurrence to be a factor for late recurrence (38). Studies on cardiac structure and function found that LAD >45 mm associated with significantly higher AF recurrence risk (39). Reduced LVEF is also linked to recurrence (40). In AF patients with impaired LVEF, ATAs post-ablation are significantly associated with increased rates of long-term adverse clinical events (9, 10). Thromboembolic events, including ischemic stroke, subclinical brain injury, mesenteric artery embolism, and renal infarction, are all associated with an increased risk of AF-related mortality (28, 41). In clinical practice, post-ablation AF recurrence is not uncommon, and the above risk factors may all influence procedural efficacy. Particularly in patients whose risk factors are not readily modifiable, more precise recurrence prediction models should be developed, with regular electrocardiographic and cardiac function monitoring to enable timely detection of recurrence and to improve maintenance of sinus rhythm and overall quality of survival.

In practice of RFCA for AF, differences associated with ICE guidance should be interpreted at the level of specific PVA segments, rather than as a simple change in the overall mean FOT. In this study, both before and after PSM, persistent FOT differences were observed in the left posterior, RAS, RPS, and RB segments. These findings suggest that ICE affects catheter-tissue contact and energy delivery, and that this effect is related to interindividual atrial structural variation and site-specific differences in catheter contact difficulty. Therefore, focusing only on global metrics across all segments is insufficient. Studies on ablation power have found recurrence sites often located at carinas between superior and inferior PVs, with sites of ablation gaps or sub-target AI values frequently at the left PV ridge and both PV roofs, attributed to catheter manipulation difficulty (42). Concerns about esophageal injury also contribute to anatomical gaps and suboptimal lesions on the posterior wall (42). To obtain optimal catheter contact, beyond changing to a steerable sheath, experienced operators often perform fine adjustments of the needle position at the FO during transseptal puncture.

Real-time ICE monitoring guides adjustment of the transseptal puncture site. However, previous researches on ICE have largely focused on reducing fluoroscopy time and fluoroscopy dose, and on shortening procedure durations and ablation times (14). A meta-analysis incorporating studies published prior to 2018 demonstrated that ICE reduced fluoroscopy time by an average of 6.95 min, alongside a significant decrease in X-ray dose (14). In 2021, Minciuna et al. verified that ICE lowered fluoroscopy dose (11,839.60 ± 6,100.6 vs. 16,260.43 ± 8,264.5 mGy, *P* = 0.041) (43). A study reported that RFCA with guidance of ICE and ultrasound-guided femoral venipuncture (USGVC) had shorten ablation time (1,686 s vs. 1,792 s, *P* = 0.012) (44). Regarding the total procedural time, opinions varied across different studies. While some research have found fewer recurrences and reduced re-hospitalization rates with ICE-guided RFCA, the full intraprocedural value of ICE was not fully explored (45). A real-world study involving 227 patients demonstrated that ICE guidance correlates with higher first-attempt success rates for atrial septal puncture during RFCA (21). A multicenter, small-sample retrospective study showed that, for operators beginning to learn radiofrequency ablation, ICE can reduce radiation exposure throughout the entire learning curve (46). ICE-guided ablation can be performed without placement of a coronary sinus electrode, a step that was previously conducted under fluoroscopy. Moreover, during transseptal puncture, ICE reduces the time adjusting for suitable puncture position. However, small single-center retrospective studies have reported differing views. Some investigators have suggested that the combination of ICE and a three-dimensional electro-anatomical mapping system enabled completely fluoroless ablation while shortening both ablation time and overall procedural time (47). Nevertheless, the reduction in total procedure duration cannot be fully explained solely by shorter transseptal puncture time or reduced fluoroscopy time. The role of ICE guidance in RFCA still requires further evaluation using broader and more refined metrics and analytical approaches. As providing clearer visualization of anatomical structures, it is needed to investigate outcome measures by anatomical subregions rather than limiting the focus to overall assessments.

Early researches on ablation parameters focused on contact force (CF), ablation duration and the influence of the force-time integral (FTI) in RFCA (39). Previous recommendations suggested a CF of at least 20 g and an FTI of at least 400 gs (11, 48, 49). Intra-procedural monitoring of CF dynamics reduces pulmonary vein reconnection and has shown clear efficacy in lowering recurrence in PeAF (50). Recent studies have focused more on interlesion distance (ILD), ablation index (AI) and ablation power. AI is influenced by factors including ablation power, CF, ablation duration and ablation temperature (51). The CLOSE protocol established targets of AI ≥550 for the anterior wall and ≥400 for the posterior wall (52). Following this as fundamental ablation strategies for AF, Hoffmann et al. further demonstrated that a pre-set ILD of 3–4 mm was superior to 5–6 mm (53). Subsequently, Chieng et al. demonstrated that a high-power, short-duration (HPSD) strategy was associated with less recurrence than a low-power, long-duration (LPLD) strategy (54). A multicenter, prospective, large-scale clinical study has shown that AI-guided RFCA for both PAF and PeAF achieves high recurrence-free rates at one-year follow-up (55). Since the introduction of the CLOSE protocol and its subsequent modifications, preset targets for indices such as FOT and ablation index (AI) have been routinely applied in radiofrequency ablation. During ablation, energy delivery at each lesion is generally terminated once the preset AI target is reached (56, 57). Despite AI attainment, abnormal FOT still needs close attention. Excessively high FOT indicates rapid temperature rise with shortened lesion ablation time and cause insufficient lesion depth, whereas excessively low FOT suggests poor catheter contact and insufficient lesion width. In an *ex vivo* experiment, investigators performed ablation on swine ventricles. Under the same ablation index (AI) setting, relatively lower CF produced greater lesion depth (58). In a clinical study, Wang et al. compared different FOT thresholds and reported that with CF >10 g and 50% stability time, nearly half of the ablation tags disappeared and a continuous encircling ablation line could not be achieved (no CEAL). Therefore, a setting of CF >5 g with 50% stability time was considered more appropriate (59). These findings suggest that in radiofrequency ablation, higher CF and higher FOT do not necessarily translate into better procedural outcomes. While operators can adjust ablation time per lesion to achieve satisfactory AI by CLOSE protocol, FOT abnormalities remain important. Compared with the large number of studies targeting various procedural metrics, research specifically focused on FOT remains limited. In this center, the optimal FOT range defined was based on institutional experience. In the VISITAG system, each ablation tag is colored according to the FOT. In prior studies, FOT settings were predominantly based on minimum CF and minimum time-in-range thresholds. Tanaka et al. predefined the target FOT as CF >5 g for >25% of the total ablation time (60). Wang et al. compared two FOT-setting strategies, CF >5 g with 50% stability time and CF >10 g with 50% stability time (59). However, regarding the upper limit of an optimal FOT range, the published literature still lack a unified standard. Therefore, under the same ablation strategy, abnormalities in FOT may better reflect the impact of ICE than AI variation alone. Given the complex mechanisms of AF recurrence and substantial inter-patient variability in intracardiac anatomy, individualized ablation strategies are of high clinical value. During PVI, the posterior wall and anterosuperior regions are strongly affected by atrial anatomy, respiratory motion, catheter stability, and shielding by intracardiac structures. These areas are more prone to insufficient or excessive contact force. The region superior to RSPV lies close to Bachmann's bundle. Abnormally elevated FOT in this region may shorten lesion delivery time and thereby increase recurrence risk. As the FO adjacent to RIPV, adequate catheter contact is more difficult to maintain. A higher FPI of right PVA suggests better catheter contact at the RPVs and potentially a more favorable FO puncture site, thereby reducing recurrence risk. Accordingly, a non-significant difference in overall FOT across a unilateral pulmonary vein ablation circle does not necessarily indicate equivalent local lesion quality. By providing real-time intracardiac imaging, ICE can improve puncture-site selection and catheter-wall contact assessment, thereby altering the distribution of local lesion quality.

After PSM, a statistically significant difference in procedural duration was observed between the two groups, whereas ablation duration did not reach. This finding suggests that the impact of ICE guidance may be more related to time allocation within the overall procedural workflow, rather than a direct effect on the ablation phase itself. In this study, procedure time was not further partitioned into specific procedural components, such as preparation time, transseptal puncture time and mapping time. In the post-PSM comparison between the two groups, the distribution of procedural time differed between the ICE and non-ICE groups, whereas subsequent correlation and regression analyses showed no clear direct association between ICE and procedure duration. These findings suggest that any effect of ICE on procedure duration may be indirect, potentially mediated by changes in FOT distribution and puncture efficiency. In this study, prolonged procedural duration was associated with a higher count of excessively high FOT at the superior portion of the left ablation circle, prior history of cardiomyopathy, and concomitant LAAO. Cardiomyopathy indicates more advanced underlying structural remodeling, with greater atrial heterogeneity, which may make restoration of sinus rhythm more difficult. The “one-stop” procedure strategy would understandably increase total procedural time. The superior segment of the LSPV is adjacent to the terminal myocardial branch (TMB), which connects the left terminal crest (LTC) and Bachmann's bundle. Local FOT abnormalities may reflect unstable catheter–tissue contact control in this region and increased ablation difficulty.

In terms of predictors of recurrence, the final multivariable model in this study retained baseline eGFR and lesions with abnormally high FOT in RPS segment. A higher eGFR was identified as a protective factor associated with a lower risk of recurrence, suggesting that renal functional status may serve as a biological marker of prognosis after AF ablation. Previous studies have indicated that chronic renal dysfunction is associated with inflammatory activation, oxidative stress, sympathetic overactivity, and atrial fibrotic remodeling, all of which may reduce post-ablation sinus rhythm maintenance (2, 36, 37). Our findings further support the value of preprocedural renal function assessment for risk stratification. An increased number of abnormally high-FOT lesions in the RPS segment was associated with a higher recurrence risk, indicating that lesion-quality abnormalities in this region may reflect impaired lesion continuity and suboptimal tissue response. As noted above, even with guidance from indices such as AI, excessively high or fluctuating contact may still compromise transmural lesion formation and leave a substrate for recurrence. In this study, Kaplan–Meier analysis showed between-group differences in recurrence curves when stratified by ICE use. However, ICE did not remain significant in the final multivariable model. This finding suggests potential interactions between ICE and other covariates, and implies that its effect may be manifested indirectly through improved local ablation efficacy and optimized spatial lesion distribution. In RFCA, ICE can be regarded as an important tool for enhancing procedural visualization and safety margins. But the benefits of ICE still depend on case complexity, the operators learning curve, ablation strategy, and comprehensive postprocedural management.

## 5 Conclusion

This study found that the guidance of ICE in RFCA influences the distribution of abnormal FOT across multiple PV segments. ICE-guided procedures demonstrated improvement in AF recurrence. However, this benefit may not be directly provided by ICE itself but rather mediated through the reduction of certain procedural characteristics, particularly the occurrence of abnormal FOT and gaps. For assessing the risk of post-AF ablation recurrence, attention should be paid not only to the patient's overall preoperative condition but also to the heterogeneity of the atrial segments. Maintaining FOT within an appropriate range is crucial for the effective creation of transmural ablation.

## 6 Limitations

This study is a single-center study, which may limit the representativeness of sample. When handling multivariate, segment-level high-dimensional parameters, there may be insufficient statistical power. The retrospective design introduces recall bias. Being retrospective, the study is subject to loss-to-follow-up bias. Recurrence might reduce patient compliance with follow-up. Patients included were those undergoing RFCA for AF during the initial implementation period of ICE at this center, which caused selection bias and information bias. Non-randomized assignment of ICE use was one of the major limitations. In this center, whether ICE was used depended on operator judgment and patient preference, which introduced potential selection bias. In this study, the operator-level factors, such as individual experience, technical preference, and case-selection behavior, were not selected into the analysis. These factors may independently affect procedural duration, lesion quality and recurrence. This preference-driven treatment allocation was a major source of potential residual bias in the study. Future research should aim to conduct multicenter prospective studies and expand the sample size. External validation and standardized procedural protocols are needed to enhance the level of evidence.

## Data Availability

The original contributions presented in the study are included in the article/Supplementary Material, further inquiries can be directed to the corresponding author/s.
